# Effects of Environmental Factors on the Performance of Ground-Based Low-Cost CO_2_ Sensors

**DOI:** 10.3390/s25196114

**Published:** 2025-10-03

**Authors:** Xiaoyu Ren, Kai Wu, Dongxu Yang, Yi Liu, Yong Wang, Ting Wang, Zhaonan Cai, Lu Yao, Tonghui Zhao, Jing Wang, Zhe Jiang

**Affiliations:** 1 Carbon Neutrality Research Center (CNRC), Institute of Atmospheric Physics, Chinese Academy of Sciences, Beijing 100029, China; 2 Key Laboratory of Middle Atmospheric Physics and Global Environment Observation (LAGEO), Institute of Atmospheric Physics, Chinese Academy of Sciences, Beijing 100029, China; 3 Nanjing ZTWEATHER Technology Co., Ltd., Nanjing 210044, China

**Keywords:** CO_2_ mole fraction measurements, calibration, low-cost sensors, long-term field observation

## Abstract

This paper presents a multivariable linear regression calibration method for non-dispersive infrared (NDIR) CO_2_ sensors in a low-cost carbon monitoring network. We test this calibration method with data collected in a temperature- and pressure-controlled laboratory and evaluate the calibration method with long-term observational data collected at the Xinglong Atmospheric Background Observatory. Compared to data collected by a high-accuracy cavity ring-down spectrometer (Picarro), the results show that a multivariable linear regression approach incorporating temperature, pressure, and relative humidity can reduce the mean absolute bias from 5.218 ppm to 0.003 ppm, with root mean square errors (RMSE) within 2.1 ppm after calibration. For field observations, the RMSE is reduced from 8.315 ppm to 2.154 ppm, and the bias decreases from 39.170 ppm to 0.018 ppm. The calibrated data can effectively capture the diurnal variation of CO_2_ mole fraction. The test of the number of reference data shows that about 10 days of co-located reference data are sufficient to obtain reliable measurements. Calibration windows taken from winter or summer provide better results, suggesting a strategy to optimize short-term calibration campaigns.

## 1. Introduction

Anthropogenic emissions of carbon dioxide (CO_2_) are a primary driver of climate change and global warming [1]. To study anthropogenic CO_2_ emissions from fossil fuel combustion, atmospheric measurements of CO_2_ mole fractions are crucial [2,3]. Ground-based CO_2_ measurements have made considerable progress in recent decades and have implemented a series of research projects [4,5,6,7], such as the European Integrated Carbon Observation System (ICOS) (https://www.icos-cp.eu/projects/icos-cities, accessed on 30 July 2025) [8], the International Atmospheric Greenhouse Gas Monitoring Network (GAW) [9], the Megacity Carbon Project [6,10], and the MegaParis CO_2_ Project [10]. These projects use high-precision cavity ring-down spectrometers (e.g., Picarro G2301: https://www.picarro.com/environmental/products/g2301_gas_concentration_analyzer, accessed on 30 July 2025) to establish observation networks and provide long-term, high-quality CO_2_ mole fractions for the assessment of urban CO_2_ emissions in Europe and America [10,11]. While these projects use instruments to measure CO_2_ mole fraction, the high-precision spectrometers are expensive to manufacture and maintain, and their higher cost limits their use in developing countries.

Previous studies demonstrated that increasing the density of measurement sites can reduce random measurement errors in CO_2_ mole fractions [12]. Some small-scale industrial areas tend to use multiple sensors to increase measurement density for detecting CO_2_ emissions [13]. To extend the observation coverage of CO_2_ mole fractions, low-cost CO_2_ sensors are being used with increasing urgency [14]. Although less accurate, Broquet et al. [15] demonstrated that increasing the number of instruments can reduce the errors in CO_2_ mole fraction measurements. Therefore, low-cost sensors with denser observation networks are needed to increase the spatial resolution of CO_2_ mole fraction measurements and contribute to estimating CO_2_ emissions [13,16].

Low-cost CO_2_ measurement sensors have been established for continuous monitoring of CO_2_ mole fractions in urban areas [16]. For example, the Berkeley Atmospheric CO_2_ Observation Network (BEACO_2_N) employs about fifty low-cost sensors to create a CO_2_ detection network in San Francisco, with distances of approximately 2 km between adjacent instruments [13,17]. Results show that BEACO_2_N has great advantages in quantifying regional CO_2_ emissions and local CO_2_ enhancements [17]. Additionally, Lee et al. [18] developed a mobile CO_2_ observation network in Vancouver with low-cost sensors, capturing a street-level CO_2_ mole fraction map that can verify city-level emission inventories. Other studies demonstrated that low-cost, denser CO_2_ observation networks can capture the distribution of CO_2_ mole fraction, showing that these networks can be used to quantify anthropogenic CO_2_ emissions with extensive coverage [3,13,16].

The key factors for the application of low-cost ground-based sensors are sensor accuracy and stability. Calibration methods are crucial for improving their accuracy [18,19,20]. Currently, data from low-cost CO_2_ sensors are calibrated by various methods. For instance, BEACO_2_N employs a pressure empirical correction method to reduce the offset from −1 to 0 and standard deviation from 1.5 ppm to 1.4 ppm [21]. This method, while simple and effective, does not fully address the combined effects of biases in temperature, water vapor pressure, and other factors. Further research on BEACO_2_N indicated the need for temperature dependence calibration to improve measurement accuracy [5]. However, this method is complex, involving drift correction in the instrument zero. In addition, a multivariable correction method has been applied to calibrate for high-performance platform NDIR CO_2_ sensors (HPP3) [3]. With this method, the sensor responses to the temperature (T), pressure (P), and water vapor pressure (e) are linear, and regular recalibrations should be performed to ensure data quality. The stability of measurements is another crucial metric for low-cost sensors. Verification experiments are necessary to confirm calibration methods and sensor performance over long-term observations. Most verifications are based on short-term measurements. Some previous studies conducted short-term verifications with concurrent Picarro instrument at the same location for a few weeks [7,22].

To overcome these challenges, this study aims to apply a calibration method to correct systematic and random measurement errors of seven low-cost sensors and evaluate their performance. We aim to test the accuracy and stability of low-cost CO_2_ sensors by synchronous observation with a high-precision Picarro instrument. The calibration and verification experiments are conducted with baseline measurement nodes of a novel Low-cost UAV Coordinated Carbon Observation Network (LUCCN). LUCCN consists of ground measurement nodes and unmanned aerial vehicles (UAVs), which are all equipped with non-dispersive infrared (NDIR) CO_2_ sensors. In this study, we focus on the LUCCN ground measurement nodes and refer to LUCCN sensors in the following description.

This paper introduces the LUCCN sensors, including the principle of observation with the instrument and the components of the instrument. Section 2 presents the calibration methods, results, and performance of the LUCCN nodes in a temperature- and pressure-controlled indoor laboratory. To verify the effectiveness of the calibration method and the performance of the LUCCN sensors, we evaluate the calibrated results of long-term measurements by comparing them with coincident data collected by a Picarro instrument in Section 3. Finally, we test the effectiveness of LUCCN by demonstrating its accuracy and precision for measuring changes in CO_2_ mole fractions.

## 2. Calibration in a Temperature- and Pressure-Controlled Laboratory

### 2.1. Instrument Configurations

**Figure 1:**
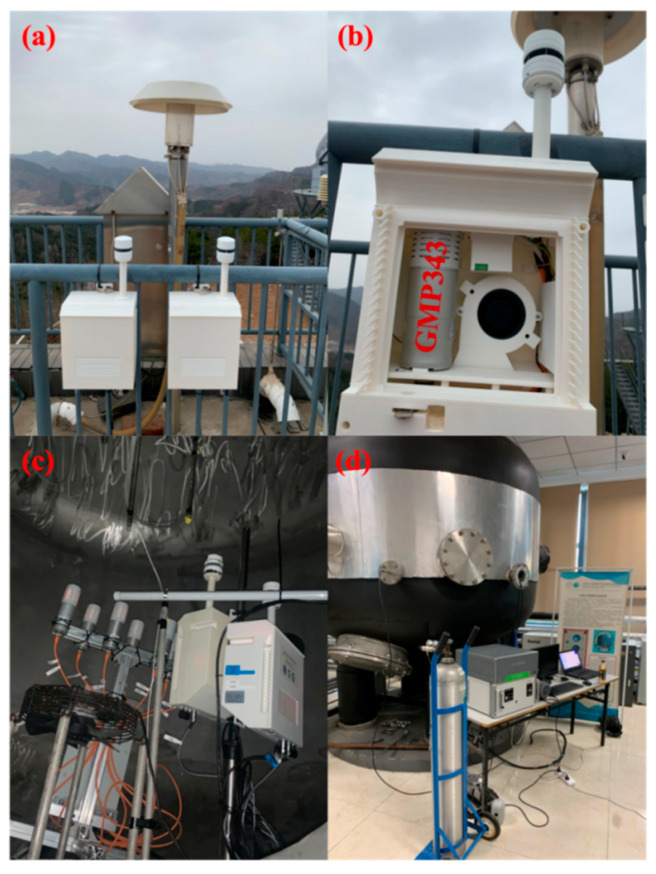
The ground-based LUCCN nodes and the diagrams of the observation fields in the laboratory: (**a**) shows the exterior diagram of the LUCCN ground station; (**b**) shows the internal structure of the LUCCN node; (**c**) shows the NDIR sensors and gas conduits in a cloud chamber where temperature, pressure, and humidity can be adjusted; and (**d**) shows the exterior of the cloud chamber, the Picarro G2301, and WMO CO_2_ standard gas cylinders.

The main component of LUCCN comprises a number of sensors that are housed in a small, newly developed weatherproof enclosure. We use a Vaisala CarboCap GMP343 (https://www.vaisala.com/en/products/instruments-sensors-and-other-measurement-devices/instruments-industrial-measurements/gmp343, accessed on 30 July 2025) for CO_2_ mole fraction measurement, as shown in Figure 1b. This sensor was introduced and tested in the BEACO_2_N project [7,17,21], showing higher stability in detecting CO_2_ mole fractions. We test the accuracy and precision of GMP343 in different environments, including varying temperature, pressure, and relative humidity. Variations in these variables affect the measurement accuracy by changing the intensity of the infrared light source and the absorption of the gas molecules. The accuracy of measurement varies between sensors due to manufacturing settings and factory calibration methods. Each node contains a slot for the installation of sensors to measure co-emitted pollutants (CO, NO_2_, PM2.5), which helps to estimate the emission sectors [23]. For example, we use CO and NO_2_ sensors from AlphaSense, which are used in greenhouse gas monitoring networks.

To correct the measurement bias caused by the environment, we integrate pressure and humidity sensors into each node. Since all chambers of the GMP343 have Pt1000 temperature sensors, external temperature sensors are unnecessary. For flux inversions, we integrate a Vaisala WXT-536 supersonic anemometer to measure wind speed and wind direction. The sensors for each node are integrated into a specially designed weatherproof housing. We optimize the design of the shutter for alternative mounting requirements. To facilitate the transmission and storage of network data, we develop a special infrastructure for the Internet of Things and a database. For data security reasons, the database software can be used on any local network without centralized storage. The total power requirement is less than 5 W, allowing us to use solar panels instead of power cables. The main specifications of the LUCCN sensors are summarized in Table 1. LUCCN sensors are more affordable to manufacture and maintain, easier to use, and more mobile. These features are crucial for enabling denser measurements of CO_2_ mole fraction.

### 2.2. Calibration and Evaluation in an Indoor Laboratory

Previous chamber tests indicate that the measurement biases are due to environmental conditions, such as pressure, temperature, and relative humidity [3,7,13,20]. Therefore, we develop a bias-correction method to reduce environment-induced errors based on the manufacturers’ standard corrections. In our calibration, each sensor was calibrated in an environmentally controlled chamber where temperature, pressure, and humidity can be adjusted (Figure 1c). The environmentally controlled chamber was provided by the Beijing Municipal Meteorological Bureau. Seven LUCCN sensors and one Picarro G2301 were used simultaneously as measurement devices. We connected a Picarro G2301 spectrometer to the environmentally controlled chamber via an airtight tube. The CO_2_ mole fraction inside the cloud chamber was varied experimentally, as shown in Figure 1d. During the calibration experiment, the CO_2_ mole fraction in the chamber varied from 422 ppm to 444 ppm, the temperature from −10 °C to 24 °C, the pressure from 978 hPa to 1032 hPa, and the relative humidity from 56% to 89%. The Picarro G2301 was calibrated every two hours with WMO standard gas (with a CO_2_ mole fraction of 402 ppm as the calibration value) to correct for possible drift of the Picarro instrument.

**Figure 2:**
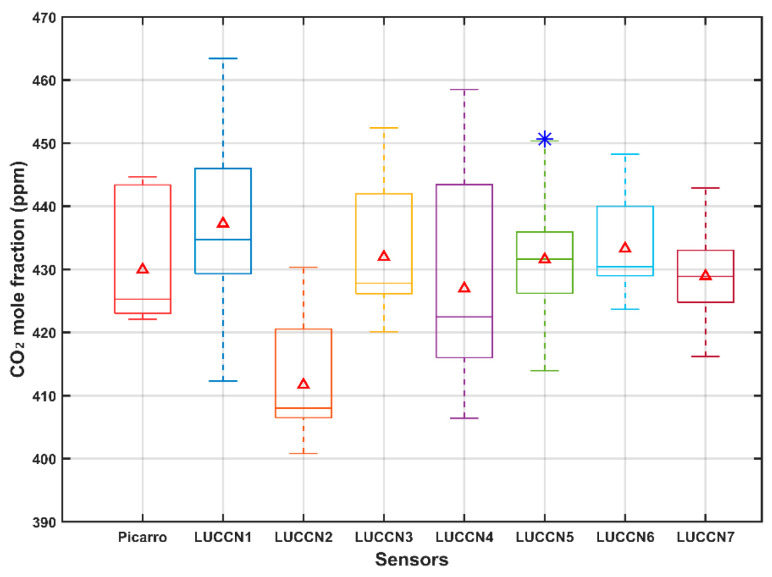
Boxplot of CO_2_ mole fraction measurements from the Picarro instrument and the seven original LUCCN sensors in the laboratory. The red triangles, lines, and asterisks represent the mean, median, and outliers, respectively. The bottom and top of the box indicate the 25th and 75th percentiles. The whiskers extend to the most extreme data points that are not considered outliers and are more than 1.5 times the interquartile range away from the top or bottom of the box.

As shown in Figure 2, the original CO_2_ mole fractions of the seven LUCCN sensors show sizable variation. The mean values range from approximately 410 to 440 ppm, and the measurement biases of about ±20 ppm from the Picarro reference are evident. In addition, the range of the median values are obvious, indicating sensor-dependent characteristics. These results show the importance of individual calibration to ensure the consistency of measurements.

**Figure A1:**
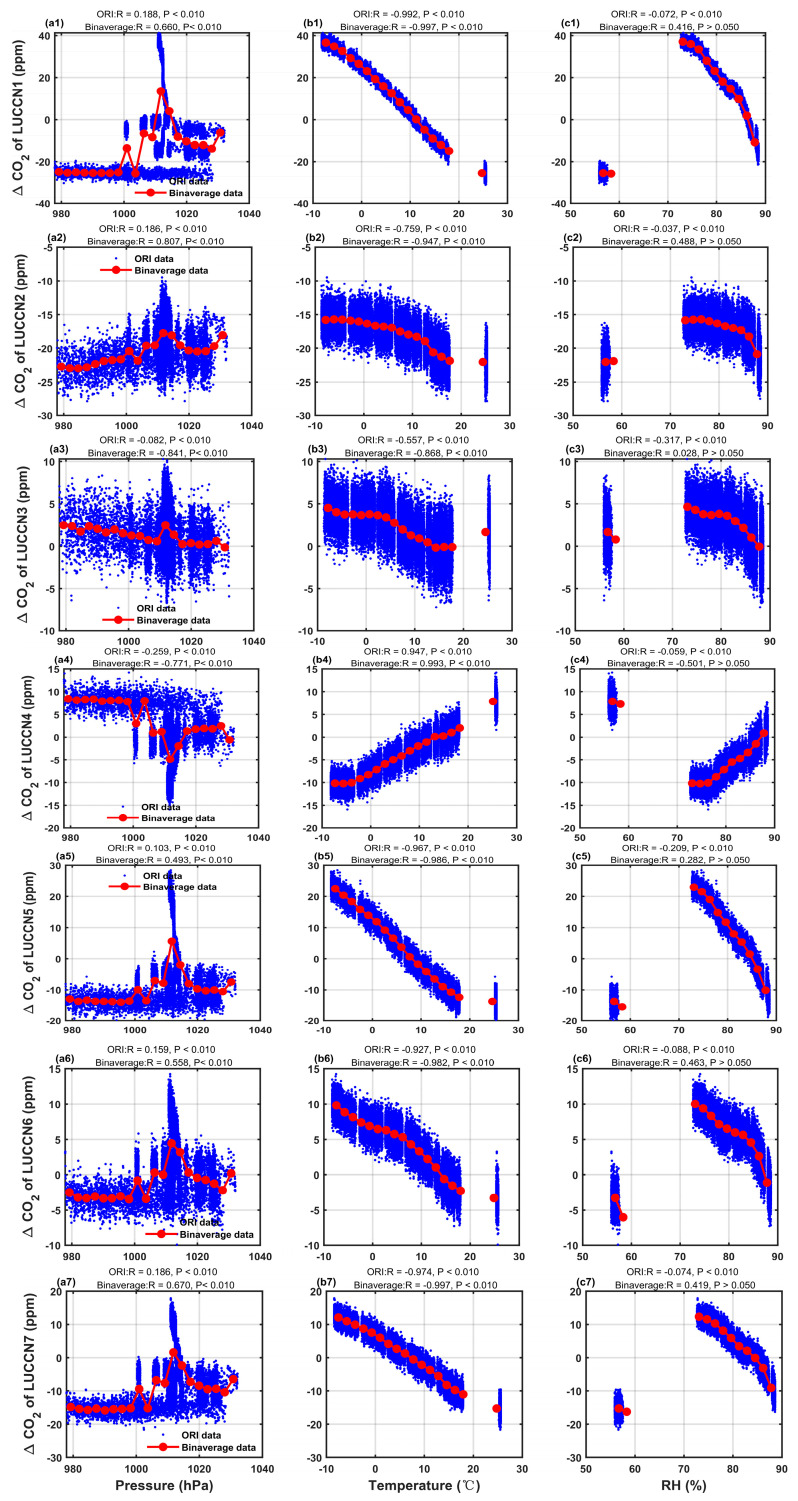
Correlation between the difference in CO_2_ mole fraction measurements (ΔCO_2_ = LUCCN–Picarro) and environmental variables (P, T, RH) during an indoor observation experiment.

For the calibration, we use a multivariable linear regression method to determine the relationship between the environmental variables and the measurement bias of CO_2_ mole fractions. In our test, we find that the changes in bias are related to pressure (P), temperature (T), and relative humidity (RH) (Figure A1). Therefore, we aim to calibrate the sensors under all possible conditions with a multivariable function. The parameters used to correct the bias vary from sensor to sensor. Therefore, we have to correct the bias of each sensor individually.

The multivariable linear regression method to correct the bias of measurement is,

(1) $${\mathrm{CO}}_{2}\;\mathrm{COR}=a\;*\;P+b\;*\;T+c\;*\;RH+e,$$

**Figure 3:**
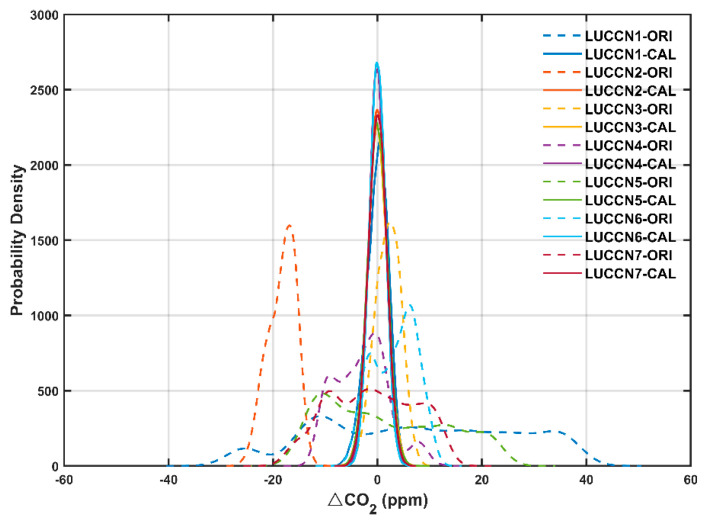
Probability density functions (PDFs) of the differences of CO_2_ mole fractions between LUCCN and Picarro (ΔCO_2_ = LUCCN–Picarro) before and after calibration in the laboratory. ORI represents original data and CAL represents calibrated data.

CO_2_ CAL = CO_2_ ORI − CO_2_ COR, (2)

where CO_2_ COR stands for the correction of the CO_2_ mole fractions of the calibration. a, b, and c are the calibration parameters for P, T, and RH, respectively, and e is the offset. We applied this method to 80% of the raw data for estimating the calibration parameters, which were then used to calibrate all the raw data. The probability density function (Figure 3) shows that, before calibration (denoted as ORI), LUCCN measurements show sizable bias and a skewed distribution compared to the Picarro measurements. After calibration (denoted as CAL), the distributions of LUCCN sensors converge to a Gaussian shape that closely matches the Picarro data, with peaks centered near 0 ppm (no bias). The reduction in bias and skewness indicates substantial improvements in systematic and random measurement errors.

The calibration parameters are listed in Table 2, which shows that both the offset and parameters vary between the sensors. The performance of calibration is evaluated in Table 3. The mean value of root mean square error (RMSE) of the seven sensors decreases from 10.347 ppm (ORI) to 1.782 ppm (CAL), while the mean absolute bias is reduced from 5.218 ppm (ORI) to 0.003 ppm (CAL). In particular, the LUCCN2 sensor, which originally shows a sizable negative bias (−18.264 ppm), reduces to a tiny bias of −0.007 ppm. In addition, the mean correlation coefficient (R) increases from 0.538 (ORI) to 0.981 (CAL). These results indicate the effectiveness of the calibration method. In Section 3, we will test the accuracy and precision of a sensor based on a long-term field observation experiment.

## 3. Field Observation Experiment

### 3.1. Observation Site and Setup

The LUCCN sensors were installed at the Xinglong Atmospheric Background Observatory site (Xinglong) of the Institute of Atmospheric Physics of the Chinese Academy of Sciences. This site is located on the summit of Lianzhai Mountain (40°24′ N, 117°30′ E) in Xinglong County, Hebei Province, China. The site is surrounded by mountains, is sparsely populated, and is hardly affected by human activities. The LUCCN sensors were installed in the immediate vicinity of the Picarro G2301 pipe inlet on the roof of the measurement platform. All sensors are placed close to each other to ensure that the measurement targets are the same, as shown in Figure 1a. We conducted the measurements from 27 October 2021, to 31 July 2022, and data were collected across four seasons, with some missing data in May 2022. The raw data included CO_2_ mole fractions, temperature, pressure, relative humidity, wind direction, and wind speed with a sampling frequency of 1 Hz measured by LUCCN. The Picarro G2301 instrument simultaneously measured CO_2_ mole fractions with a sampling frequency of 0.2 Hz.

### 3.2. Data Processing and Calibration

Unlike the indoor experiment, the responses of LUCCN sensors in outdoor field observatories are influenced by various complex variables. Therefore, data quality control before calibration is needed to improve the accuracy of LUCCN. Firstly, it is necessary to select the synchronous data of LUCCN and Picarro due to some missing records from Picarro. Secondly, we interpolated the data of LUCCN and Picarro to the same sampling frequency (1 Hz). Thirdly, we excluded the obviously anomalous data which were larger than three times the standard deviation. Finally, these data are averaged over one hour.

**Figure 4:**
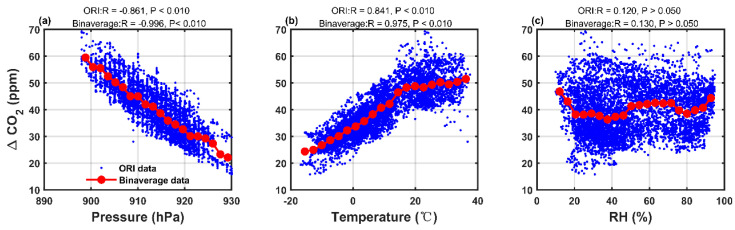
Correlation between the difference in CO_2_ mole fraction measurements (ΔCO_2_ = LUCCN–Picarro) and (**a**) pressure, (**b**) temperature, (**c**) relative humidity (RH) during an outdoor field observation experiment from 27 October 2021, to 31 July 2022.

**Figure 5:**
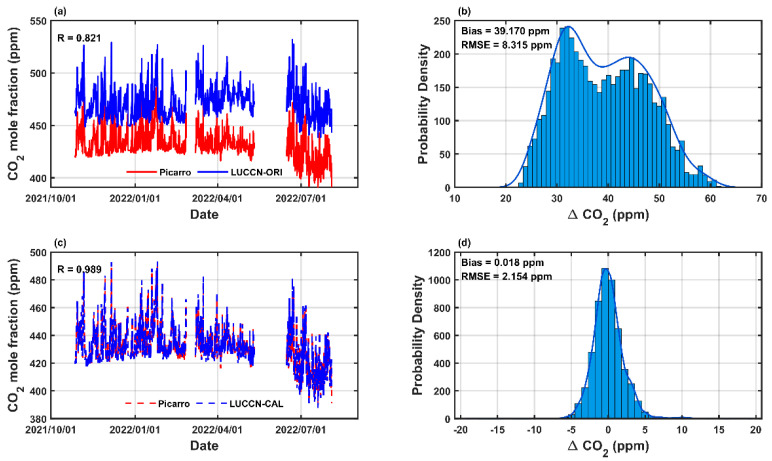
Picarro and LUCCN CO_2_ mole fraction measurements before and after calibration during an outdoor field observation experiment from 27 October 2021, to 31 July 2022. (**a**) Original CO_2_ mole fraction measurements from Picarro (red) and LUCCN (blue); (**b**) PDF of ΔCO_2_ (LUCCN–Picarro) before calibration, with bias and root mean square error (RMSE); (**c**) Calibrated CO_2_ mole fractions of LUCCN (blue) and Picarro (red); (**d**) PDF of ΔCO_2_ (LUCCN–Picarro) after calibration.

As shown in Figure 4, the CO_2_ mole fraction differences (ΔCO_2_ = LUCCN–Picarro) are correlated with environmental factors: a significant negative correlation with pressure (R = −0.861) and a positive correlation with temperature (R = 0.841). The correlation with RH is insignificant. These results indicate that the LUCCN sensors are environmentally dependent. Figure 5a shows the hourly averaged data from a LUCCN sensor and Picarro. There is a systematic bias of 39.170 ppm in the original LUCCN data, which are shown in Figure 5b. The distribution is wide and asymmetrical, indicating a significant bias in the original LUCCN measurements.

To correct for the measurement errors, the multivariable linear regression method as shown in Equations (1) and (2) is used. Firstly, the entire ranges of observed P, T, and RH in the raw dataset were divided into bins with a step size of 0.1 units (i.e., 0.1 hPa for pressure, 0.1 °C for temperature, and 0.1% for RH), bounded by the minimum and maximum values of each variable. Secondly, two observations were randomly sampled within each bin. For each sampling time point, the corresponding CO_2_ of LUCCN and Picarro, and the concurrent P, T, and RH values were recorded. The recorded data accounted for about 30% of the total data. This bin-wise sampling strategy was developed to ensure a balanced coverage of the entire spectrum of environmental conditions.

Table 4 lists the parameters of the calibration method. The comparison after calibration is shown in Figure 5c, which shows the calibrated CO_2_ mole fraction of LUCCN is consistent with the Picarro measurements. The correlation coefficient (R) increases from 0.821 to 0.989. The distribution of ΔCO_2_ after calibration is unbiased and exhibits less dispersion shown in Figure 5d, with bias decreasing from 39.170 to 0.018 ppm and RMSE reducing from 8.315 to 2.154 ppm.

**Figure 6:**
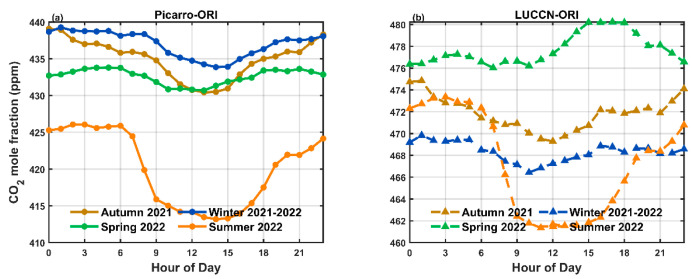
Seasonally averaged diurnal variations of CO_2_ mole fractions from the original (**a**) Picarro and (**b**) LUCCN data before calibration.

**Figure 7:**
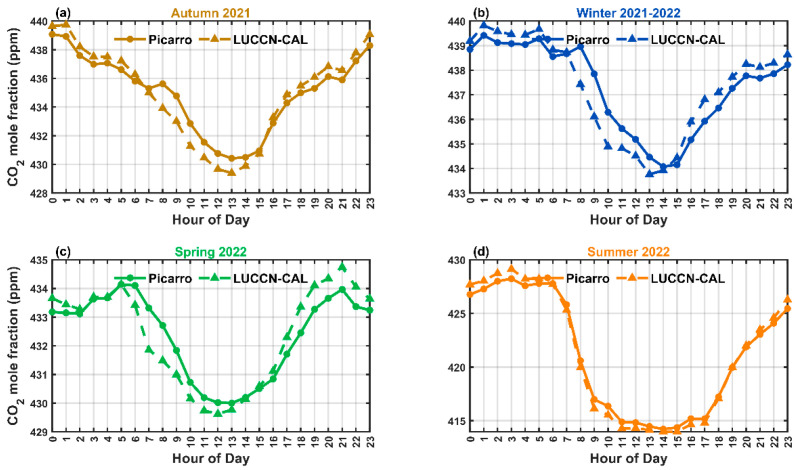
Seasonally averaged diurnal variations of CO_2_ mole fractions from the calibrated LUCCN and Picarro measurements in (**a**) autumn, (**b**) winter, (**c**) spring, and (**d**) summer from 2021 to 2022.

Figure 6 and Figure 7 show the seasonally averaged diurnal variation of CO_2_ mole fractions before and after calibration, respectively. In the original data (Figure 6), LUCCN overestimates the nocturnal and daytime CO_2_ mole fractions in all seasons. After calibration (Figure 7), LUCCN shows similarity in the amplitude of the diurnal cycles observed by Picarro, with slightly differences in the phase of autumn, winter, and spring.

### 3.3. Sensitivity Test of the Number of Calibration Data

Although the calibration method described above uses about 30% of Picarro data to derive the environment-based calibration parameters, such long-term reference data are not always available in practice. Therefore, it is necessary to evaluate how much of the calibration data, which is the duration of the joint observation of LUCCN and Picarro, are needed to ensure the accuracy and precision of calibration when applied to an outdoor field measurement experiment.

To address this issue, a sensitivity test on the number of reference data is performed. For each season, subsets of LUCCN and Picarro data with a duration of 1 to 30 days were extracted. Unlike the previous bin-average sampling strategy, a direct time-window approach is used: For a given duration (e.g., five days), all data points within this window were used to fit the multivariable linear regression. The calibration parameters derived from each n-day subset (1 ≤ n ≤ 30) were then fitted to the entire data of LUCCN. The calibrated LUCCN data were compared to Picarro based on two metrics: absolute bias (Abs Bias) and RMSE. This procedure is repeated for each seasonal subset to account for possible seasonal dependency in calibration.

**Figure 8:**
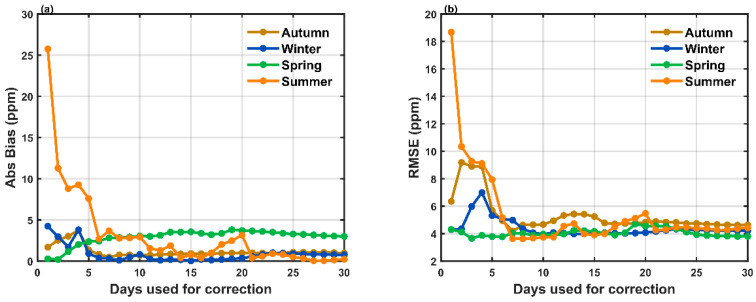
(**a**) Absolute bias (Abs Bias) and (**b**) root mean square error (RMSE) for different durations of reference data for calibration in different seasons.

Figure 8 shows the impact of the number of reference data on the measurement accuracy and precision. Both Abs Bias and RMSE decrease significantly with increasing calibration data up to seven days. Beyond 10 days, the metrics begin to stagnate, and extending the calibration period from 10 to 30 days yields minor improvements. Calibration in autumn shows a slightly higher RMSE, and calibration in spring shows a slightly higher Abs Bias, likely due to the relatively narrow ranges of P, T, and RH in these seasons. These results suggest that using at least 10 days of reference data for calibration can reduce systematic and random measurement errors in the LUCCN measurements, especially when using reference data from winter and summer.

These results provide practical guidance for field observations: it is recommended to run at least 10 days of joint LUCCN and Picarro measurements to ensure year-round calibration. Using reference data from seasons with higher environmental variability (e.g., winter or summer) can reduce biases and RMSE in LUCCN calibration. In previous studies, ‘medium-accuracy’ refers to CO_2_ sensors whose random measurement errors are less than ±5 ppm after environmental correction or periodic calibration [24,25]. Therefore, these results suggest that the LUCCN sensor, when properly calibrated, can provide medium-accuracy CO_2_ measurements in outdoor environments, supporting its application in dense observation networks for urban and regional CO_2_ monitoring.

## 4. Conclusions and Discussion

This study presents the design, calibration, and long-term evaluation of a low-cost CO_2_ mole fraction measurement system, the LUCCN sensor nodes, designed to enhance spatial and temporal resolution of regional CO_2_ monitoring. In the indoor laboratory experiment, seven LUCCN sensors were tested against a high-accuracy Picarro instrument under controlled temperature, pressure, and humidity conditions. Results indicate that the biases in LUCCN sensors are environmental dependent. A multivariable linear regression approach incorporating T, P, and RH can reduce the mean absolute bias from 5.218 ppm to 0.003 ppm for all sensors, with post-calibration RMSE values within 2.1 ppm. The mean correlation coefficient (R) increases from 0.538 to 0.981. These results affirm the feasibility of the environment-based calibration method for LUCCN sensors.

In the outdoor field observation experiment, a year-long co-location observation at the Xinglong Atmospheric Background Observatory enables the assessment of LUCCN under field conditions. The LUCCN data show strong environmental dependence, particularly on temperature and pressure. After calibration, the RMSE is reduced from 8.315 ppm to 2.154 ppm, and the bias decreases from 39.170 ppm to 0.018 ppm, which means that the accuracy of LUCCN meets the requirement of medium precision. The calibrated LUCCN data can capture diurnal variations in seasonally averaged CO_2_. The test of the number of reference data show that about 10 days of co-located data are sufficient to achieve reliable measurements. This highlights the necessity of laboratory-based calibration, which can cover a broader range of temperature, pressure, and relative humidity, ensuring that the derived parameters are robust and can reduce measurement biases.

This work suggests that these low-cost sensors, when properly calibrated, can provide reliable CO_2_ mole fraction measurements. Combined with UAV-based vertical profiling, LUCCN has the potential to form dense, three-dimensional CO_2_ observations, enabling more coverage in regions lacking expensive monitoring infrastructure. On the one hand, LUCCN ground nodes are able to conduct long-term and continuous measurements of CO_2_ mole fractions and compensate for the lack of satellite measurements. On the other hand, LUCCN nodes and UAVs form a three-dimensional observation network that can be used for joint measurements of CO_2_ mole fractions from air and ground [26]. The LUCCN system can increase the spatial and temporal coverage of CO_2_ mole fraction measurements, especially in cases involving the detection of small, rapidly changing sources and sinks [27]. Future efforts can focus on cross-site calibration transferability, real-time environmental correction algorithms, and integration with atmospheric inversion models to understand the ability of using these data to estimate local CO_2_ fluxes.

## Figures and Tables

**Table 1:** Main specifications of the LUCCN sensors.

LUCCN Sensor	Parameter
Weight	8 kg
Dimensions	83 × 31.2 × 19.5 cm
Power Consumption	<5 W
Sampling Frequency	1 Hz
Temperature Range	−52 °C–+60 °C
Pressure Range	500–1100 hPa
Relative Humidity Range	0–100%
Wind Speed Range	0–75 m/s

**Table 2:** Calibration parameters for the seven LUCCN sensors in the laboratory.

	LUNCN1	LUCCN2	LUCCN3	LUCCN4	LUCCN5	LUCCN6	LUCCN7
a	−0.001	0.027	−0.013	−0.003	0.017	−0.006	0.011
b	−2.077	−0.232	−0.156	0.550	−1.268	−0.442	−0.905
c	−0.139	−0.021	−0.088	−0.045	−0.289	−0.037	−0.070
e	35.618	−42.309	23.635	−1.093	17.448	16.156	0.716

**Table 3:** Bias, root mean square error (RMSE), and correlation coefficient (R) before (ORI) and after (CAL) calibration for the seven LUCCN sensors in the laboratory.

	Bias (ppm)		RMSE (ppm)		R	
	**ORI**	**CAL**	**ORI**	**CAL**	**ORI**	**CAL**
**LUCCN1**	7.292	0.004	19.673	2.084	−0.567	0.974
**LUCCN2**	−18.264	−0.007	18.466	1.767	0.971	0.981
**LUCCN3**	2.007	0.000	3.155	1.875	0.967	0.979
**LUCCN4**	−2.997	0.001	5.922	1.606	0.970	0.985
**LUCCN5**	1.606	−0.002	11.663	1.853	−0.034	0.980
**LUCCN6**	3.324	−0.002	5.337	1.536	0.933	0.986
**LUCCN7**	−1.035	0.004	8.216	1.756	0.455	0.981

**Table 4:** Calibration parameters for the LUCCN sensor during an outdoor field observation experiment from 27 October 2021, to 31 July 2022.

a	b	c	e
1.168	−0.2034	0.042	−1106.271

## Data Availability

The data are available upon request from the authors (renxiaoyu@mail.iap.ac.cn and yangdx@mail.iap.ac.cn).
